# Assessment of perioperative anxiety levels at three time-points during hospital stay in patients undergoing elective surgery

**DOI:** 10.1186/s13741-025-00504-0

**Published:** 2025-03-12

**Authors:** Priya Goyal, Prisha, Joshua S. Chacko, Aman Goyal, Shikha Gupta, Suneet Kathuria

**Affiliations:** 1 Department of Anaesthesiology, Dayanand Medical College and Hospital, Ludhiana, Punjab India; 2 Adesh Medical College and Hospital, Kurukshetra, India; 3 Department of Internal Medicine, Fathers Muller College, Mangalore, India; 4 Department of Internal Medicine, Seth GS Medical College and KEM Hospital, Mumbai, India

**Keywords:** Preoperative anxiety, Postoperative anxiety, Perioperative anxiety, Preoperative assessment, Patient education, Postoperative pain

## Abstract

**Background:**

Perioperative anxiety is associated with complications during and after surgery, resulting in prolonged hospital stays, and long-term physical and cognitive decline. A prospective observational study was conducted to assess anxiety levels at three time-points and identify sociodemographic factors influencing it.

**Methodology:**

Three assessments were conducted on 105 patients (18–65 years) undergoing elective surgery after informed consent: A1 (day before surgery) using the State-Trait Anxiety Inventory (STAI-Trait) form, STAI-State form, and demographic data collection; A2 (on the day of surgery) with the STAI-S2 form; and A3 (24 h post-surgery) with the STAI-S3 form and a questionnaire on information requirements and pain.

**Results:**

Average state anxiety scores were S2 (18.06) > S1 (17.55) > S3 (16.38). The primary concerns were fear of feeling pain after surgery (41%), fear of the results of the surgery(33.3%), and concerns about family (32.40%). Unmarried individuals had significantly higher anxiety scores than married individuals in S1 (20.80 vs. 16.79, *p* − 0.009) and S2 (23.10 vs. 16.87, *p* − 0.001). Females consistently scored higher than males, with a significant difference in S2 (19.51 vs. 16.79, *p* − 0.05). Patients with a medical history showed the highest anxiety in S3 (18 vs. 15.67, *p* − 0.037). Skilled workers displayed the highest anxiety levels in S1 (20.20) and S2 (22.40, *p* − 0.044) as compared to other groups, while professionals showed the highest anxiety in S3 (18.05). Females (33%), rurals (29%), and ≤ 8th-grade education group (54.5%) were significantly more likely to report receiving inadequate information about surgery compared to males (12.5%, *p* − 0.018), urbans (13%, *p* − 0.036), and higher education group (18%, *p − *0.022). Younger individuals of < 30 years (47%) were more likely to feel that more information about surgery would have relaxed them compared to 41–50 age group (7.14%, *p* − 0.016).

**Conclusion:**

The anxiety levels fluctuated over three time-points and were influenced by demographic, cultural, and psychological factors. Therefore, anxiety should be identified both preoperatively and postoperatively through an individualized approach. Additionally, a significant proportion of the population still requires more information, and the diverse informational needs across the groups underscore the necessity for individualized interviews to ascertain specific information requirements, thereby preventing any paradoxical increase in anxiety due to inappropriate information delivery.

**Supplementary Information:**

The online version contains supplementary material available at 10.1186/s13741-025-00504-0.

## Background

Perioperative anxiety encompasses a subjective feeling of uneasiness and discomfort about something unlikely to happen, experienced by the patients before, during, and after the surgery. This, in turn, can lead to a range of physiological and psychological changes including fatigue, muscular tension, and abnormal hemodynamics (Shawahna et al. 2023; Woldegerima Berhe et al. 2022) which can further cause challenges such as difficult venous access, increased demand for anesthesia due to delayed jaw relaxation, blood pressure fluctuations, arrhythmias, cerebral vascular accidents, increased postoperative pain, increased risk of infections, impaired wound healing, and cognitive decline. These factors ultimately lead to delayed recovery, impaired physical function, and increased healthcare costs (Majumdar et al. 2019; Tadesse et al. 2022a, b; Gouin et al. 2012; Maranets et al. 1999; Daoud et al. 1999; Alkan Kayhan et al. 2022; Ekinci et al. 2017; Ma et al. 2021; Tasbihgou et al. 2021).

Studies indicate that the prevalence of preoperative anxiety varies by geographical region, with the highest prevalence in Africa and Asia (54%) and the lowest in North America (24%) and Latin America (25%), attributed to differences in study designs, sociodemographic factors, and healthcare services (Semagn Mekonnen et al. 2020). There are many subjective scales such as the State-Trait Anxiety Inventory (STAI), the Amsterdam Preoperative Anxiety and Information Scale (APAIS), and the Hospital Anxiety and Depression Scale (HADS) to measure anxiety (Zemła et al. 2019). The STAI questionnaire enables the assessment of the patient’s trait and current state both preoperatively and postoperatively. Additionally, shorter versions of the STAI scale have demonstrated consistent results across diverse ethnic populations (Tluczek et al. 2009; Julian et al. 2011; Boker et al. 2002).

Given the sociodemographic variability in anxiety prevalence, further regional studies are warranted. Moreover, a few or no studies have evaluated variations in anxiety levels from the preoperative to postoperative periods across three distinct follow-up periods. This prospective observational study aimed to assess anxiety levels at three perioperative stages and identify the factors influencing these variations. The insights gained could enhance patient identification and facilitate stratification for improved anxiety management, thereby potentially leading to better outcomes.

## Methods

### Study type

This observational study was conducted under the Department of Anesthesia of a tertiary care hospital, following approval from the Institutional Ethics Committee. The study was registered in the Clinical Trial Registry—India (REF/2023/07/070715). Informed written consent was obtained from all the patients included in the study.

### Selection criteria

**Inclusion criteria:** Patients aged 18–65 years of either gender scheduled for elective surgery.

**Exclusion criteria:** Patient refusal, uncooperative patients, pregnant women, patients with psychiatric illness and cognitive deficits.

### Instruments

The study used the short version of STAI (License obtained from the Mind Garden Publishers for the original and translated versions) for anxiety measurement and a self-designed questionnaire to assess reasons for feeling anxious and feedback on pain and information requirements. The questionnaire was provided to patients in three languages according to their preferences (English, Hindi, and Punjabi). The Short version of the STAI form comprises ten questions each for the State and Trait anxiety, extracted from the full version, which contains 20 questions for each type. This has a 4-point Likert scale scoring system based on the response options “not at all” to “very much so” for state anxiety and “almost never” to “almost always” for trait anxiety. Higher scores indicate higher levels of anxiety. The self-designed questionnaire was divided into two parts: the first section collected sociodemographic data and reasons for anxiety during the initial visit, while the second section focused on postoperative pain and informational feedback gathered 24 h after surgery.

### Procedure

Patients scheduled for elective surgery were evaluated for eligibility in the evening before their operation in the general surgery, orthopedics, and urology wards after their pre-anesthetic checkup. The study’s objectives and process for completing the STAI and the custom questionnaire were explained to the patients. The initial assessment (A1) involved completing the STAI-S1, STAI-T, and first section of the questionnaire. As part of the hospital protocol, one tablet of alprazolam (0.25 mg) was administered to all patients at night before the scheduled surgery and in the morning 2 h before admission to the preoperative holding area. On the day of surgery, the A2 assessment was conducted in the preoperative area, during which the patients completed the STAI-S2 form. The last assessment (A3) took place 24 h post-surgery after assessing their consciousness level, where patients completed the STAI-S3 and the second part of the questionnaire on information requirements and pain levels.

### Data synthesis

Frequency tables were generated for age, sex, marital status, occupation, education level, type of surgery, past surgery exposure, and past medical history. A two-sided chi-square test was used to determine the statistical significance of the associations between anxiety and the sociodemographic variables. A *p* value of < 0.05 was considered statistically significant. To compare the anxiety levels preoperatively and postoperatively, paired *t*-tests were used.

### Sample size calculation

According to a systematic review and meta-analysis by Semagn Mekonnen et al. (2020), the global pooled incidence of preoperative anxiety in middle- to low-income countries is 48% (Semagn Mekonnen et al. 2020).

Given confidence level = 95% and precision (*d*) = ± 10%, the formula used was.

*n* = (*Z*2*α* × *P* × (1-*P*))/*d*2,

where.

*Zα* is the value of the standard normal variate corresponding to the *α* level of significance,

*P* is the likeliness of the parameter,

*Q* is the 1 – P, and.

*d* is the margin of error, which is a measure of precision).

Based on these assumptions, the required sample size was determined to be 96. Therefore, 105 patients were included in the study for a dropout rate of 10%.

## Results

The participants were not evenly distributed in smoking history, alcohol history, and illicit substance use history, which could affect the reliability of the results and, therefore, were not further studied here as shown in Table 1.

**Table 1:** The sociodemographic characteristics of the study population

Category	Variables	No. of cases	Percentage (%)
Age group (years)	< 30	19	18.1
	31–40	31	29.5
	41–50	28	26.7
	> 50	27	25.7
Gender	Female	49	46.7
	Male	56	53.3
Marital status	Married	85	81.0
	Unmarried	20	19.0
Residence	Urban	46	43.8
	Semi-urban	19	18.1
	Rural	40	38.1
Education level	Classes 10 to 12	45	42.9
	Classes 8 and less	11	10.5
	Graduate	49	46.7
Occupation	Arithmetic skill jobs	36	34.3
	Professional	21	20.0
	Skilled worker	11	10.5
	Unemployed	37	35.2
Past medical history of any chronic diseases	No	73	69.5
	Yes	32	30.5
Past surgical history	No	49	46.7
	Yes	56	53.3
Smoking history	No	100	95.2
	Yes	5	4.8
Alcohol history	No	94	89.5
	Yes	11	10.5
Illicit Substance use history	No	103	98.1
	Yes	2	1.9

The mean state anxiety score trend was S2 > S1 > S3. Age was negatively correlated with STAI-T, S1, and S2. There was no significant correlation with the S3 score, pain level, or surgery duration, suggesting that the younger population is not a risk factor for higher postoperative anxiety or pain. The STAI S1 was strongly positively correlated with STAI S2 and moderately correlated with STAI S3. The STAI S2 was moderately positively correlated with the STAI S3, which in turn was moderately positively correlated with self-reported pain. Self-reported pain also showed a moderate positive correlation with the duration of surgery as shown in Table 2.

**Table 2:** Mean values of the STAI scores at three time-points and their correlations

Variable	Correlates with	Pearson correlation	*P* value
Age in years 41.62 ± 11.81 (18 − 65)	STAI T	− 0.335	0.001
	STAI S1	− 0.278	0.004
	STAI S2	− 0.297	0.002
STAI T 17.7 ± 5.11 (11 − 31)	STAI S1	0.575	0.001
	STAI S2	0.486	0.001
	STAI S3	0.472	0.001
STAI S1 17.55 ± 6.24 (10 − 37)	STAI S2	0.824	0.001
	STAI S3	0.487	0.001
STAI S2 18.06 ± 7.12 (10 − 33)	STAI S3	0.505	0.001
STAI S3 16.38 ± 5.27 (10 − 33)	Pain	0.4	0.001
Pain 3.5 ± 2.22 (0–8)	Duration of Surgery	0.349	0.001

In further subgroup analysis of responses received in Table 3, we found associations between residence and education with two of the mentioned concerns in Table 3. Rural dwellers (57.7%) were more likely to report fear of feeling pain during surgery than their counterparts (urban, 23.1%; suburban, 19.2%, p value—0.032). Those with 10 to 12-grade education levels (46.2%) were more likely to report fear of feeling pain during surgery as compared to graduates (30%, p value—0.027) and classes 8 or less (23.1%, p value–0.027). Those with 10 to 12-grade education levels (44.4%) were more likely to report fear of something gone wrong as compared to graduates (33.3%) and class 8 or less (22.2%, p value–0.045). The rest of the fears did not reveal any statistical significance for any of the demographic variables.

**Table 3:** Responses to the questionnaire on reasons for feeling anxious

Which of the following is bothering you	No. of cases	Percentage (%)
Fear of feeling pain after surgery	43	41.0
Fear of results of surgery	35	33.3
Concern about family	34	32.4
Fear of something gone wrong	27	25.7
Fear of feeling pain during surgery	26	24.8
None	24	22.9
Fear of unknown	21	20.0
Waking up during surgery	12	11.4
Financial burden	12	11.4
Others (fear of needle, fear of delayed recovery and fear of blood)	3	2.85

1. In Table 4, females consistently had greater STAI scores than males for S1, S2 (*p* = 0.05), and S3. The female anxiety levels were as follows: S2 > S1 > S3. In contrast, the male anxiety levels exhibited an S1 > S2 > S3 trend.
2. The < 30 years age group reported the highest mean anxiety scores across all STAI measures, with statistical significance at T (*p* value = 0), S1 (*p* value = 0.04), and S2 (*p* value = 0.006). The 41–50 age group had the lowest mean scores for T and S3, with moderate scores for S1 and S2. Finally, participants older than 50 years had the lowest mean anxiety scores at S1 and S2, with moderate mean scores at S3. The difference was statistically significant for S1 and S2 but not for S3. All age groups exhibited the pattern S2 > S1 > S3, except for the older age group which exhibited an S3 > S1 > S2 trend.
3. Unmarried people had statistically significantly greater STAIs than married people for T (*p* value = 0.001), S1 (*p* value = 0.009), and S2 (*p* value = 0.001) but not for S3. Both the groups followed the pattern of S2 > S1 > S3.
4. For *T*, S1, and S2, unemployed and semi-skilled workers had the highest levels of anxiety, professionals stood in the middle, and those with arithmetic skills had the lowest scores. However, for S3, professionals had the highest anxiety level, and skilled workers had the lowest anxiety level (a statistically significant difference in S2 (p value = 0.03), but not in S1 or S3). Semi-killed, skilled, and unemployed individuals followed the S2 > S1 > S3 pattern. The arithmetic job strata followed the S1 > S2 > S3 pattern, whereas the professional job strata followed the S2 > S3 > S1 pattern.
5. Patients with a past medical history had significantly higher STAI S3 scores (p value = 0.03) than those without a past medical history. Individuals with a past medical history had S3 > S1 > S2. However, individuals without a medical history had S2 > S1 > S3.
6. Patients who underwent regional anesthesia had consistently higher anxiety scores than those who underwent general anesthesia at T, S1, S2, and S3, although the difference was not statistically significant. Both groups followed the pattern of S2 > S1 > S3.

**Table 4:** Analysis of the effects of sociodemographic variables on perioperative anxiety levels

	Variable	STAI T	STAI S1	STAI S2	STAI S3
Gender	Female	17.71	18.16	19.51	17.20
	Male	17.70	17.02	16.79	15.66
	*P* value	0.986	0.35	**0.05**	0.135
Age (years)	< 30	20.16	20.47	22.53	16.89
	31–40	19.26	17.65	18.06	16.74
	41–50	15.75	17.68	17.79	15.86
	> 50	16.22	15.26	15.19	16.15
	*P* value	**0**	**0.047**	**0.006**	0.887
Marital status	Married	16.85	16.79	16.87	15.89
	Unmarried	21.35	20.80	23.10	18.45
	*P* value	**0.001**	**0.009**	**0.001**	0.51
Residence	Urban	16.8	16.72	16.48	16.74
	Sub-urban	17.16	17.63	19.16	15.58
	Rural	19.00	18.48	19.35	16.35
	*P* value	0.121	0.431	0.133	0.725
Education	Classes 8 and less	18.55	20.00	21.45	18.18
	Classes 10 to 12	17.84	16.64	17.18	16.02
	Grauation	17.39	17.84	18.10	16.31
	*P* value	0.774	0.255	0.204	0.477
Occupation	Arithmetic Jobs	16.58	15.81	15.39	15.11
	Professional	17.90	17.81	18.43	18.05
	Skilled worker	18.18	18.36	19.73	15.18
	Unemployed	18.54	18.86	19.95	17.03
	*P* value	0.42	0.197	**0.037**	0.15
PMH	No	17.51	17.99	18.12	15.67
	Yes	18.16	16.56	17.91	18.00
	*P* value	0.551	0.284	0.886	**0.037**
PSH	No	18.00	18.65	19.31	16.41
	Yes	17.45	16.59	16.96	16.36
	*P* value	0.582	0.091	0.093	0.961
Anesthesia	General	17.49	17.26	17.87	16.09
	Regional	18.11	18.11	18.42	16.94
	*P* value	0.559	0.51	0.71	0.432

*PMH* Past medical history of any chronic illness, *PSH* Past surgical history—any past surgery under general or local anesthesia; anesthesia—type of anesthesia being given for current surgery

On further analysis of the four questions as shown in Table 5, we found that

1. Feeling adequately informed or uninformed about anesthesia did not correlate with anxiety scores at T, S1, S2, or S3.
2. Individuals who believed that more anesthetic information would be relaxing had significantly greater anxiety than those who did not at T1 (yes: 19.31, no: 16.90; p = 0.022). Similarly, S1 and S2 also exhibited non-significant patterns (S1:17.40 “No” vs. 17.86 “Yes”; S2:17.30 “No” vs. 19.57 “Yes”; S3:16.51 “No” vs. 16.11 “Yes”).
3. Next, the group feeling inadequately informed about surgery had significantly greater S2 anxiety scores than did their informed counterparts (S2:16.91 “No” vs 22.3 “Yes,” p value = 0.002). However, the other scores were not significantly different but showed similar patterns (STAI-T: 18.39 “No” vs. 17.51 “Yes,” S1:19.26 “No” vs. 17.07 “Yes,” S3:16.65 “No” vs. 16.30 “Yes”).
4. Regarding the last question, individuals who believed that more surgical information would be relaxing exhibited consistently higher anxiety scores than those who disagreed, although the difference was not statistically significant (T: 17.23 “No” vs. 18.90 “Yes” (p = 0.130), S1: 17.33 “No” vs. 18.10 “Yes” (p = 0.572), S2: 17.55 “No” vs. 19.33 “Yes” (p = 0.247), and S3: 16.40 “No” vs. 16.33 “Yes” (p = 0.954)).

**Table 5:** Responses to the questionnaire on information requirements

Information feedback questionnaire	Response	No. of cases	Percentage (%)
Do you think that you were given adequate information about anesthesia	No	28	26.7
	Yes	77	73.3
Do you think if you were given more information about anesthesia, it would have made you more relaxed	No	70	66.7
	Yes	35	33.3
Do you think that you were given adequate information about surgery ?	No	23	21.9
	Yes	82	78.1
Do you think if you were given more information about surgery, it would have made you more relaxed?	No	75	71.4
	Yes	30	28.6

On further subgroup analyses of patient perceptions across sociodemographic parameters revealed that females (33%), rural dwellers (29%), and those with ≤ 8th-grade education (54.5%) were significantly more likely to report receiving inadequate information about surgery than males (12.5%, *p* = 0.018), urban residents (13%, *p* = 0.036), and those with higher education (18%, *p* = 0.022). Younger individuals (47%) were more likely to feel that more information about surgery would have relaxed them than 41- to 50-year-old individuals (7.14%, *p* = 0.016). Other variables such as marital status, occupation, type of anesthesia, and medical or surgical history did not significantly affect any of the four questions.

## Discussion

The results of the present study showed that anxiety was highest on the day of surgery, followed by the day before surgery and then the day after surgery (i.e., S2 > S1 > S3), consistent with prior research indicating higher preoperative anxiety levels (Akinsulore et al. 2015; Kumar et al. 2019; Nijkamp 2004; Reyes-Gilabert 2017; Pokharel et al. 2011). However, some studies have not observed an increasing trend in preoperative anxiety as surgery approaches, which may be attributed to varying sociodemographic factors and study designs (Badner et al. 1990). The postoperative decrease in anxiety in our study can be attributed to the alleviation of symptoms and resolution of misconceptions about surgical or anesthesia outcomes due to misinformation from the public. Additionally, despite administering premedication between the S1 and S2 assessments as a part of the routine hospital protocol, patients’ anxiety continued to rise until S2, indicating that premedication alone may be insufficient. This highlights the need to incorporate other interventions, such as non-pharmacological interventions, in the form of music therapy or meditation. Future research could explore the efficacy of combining pharmacological and non-pharmacological approaches to mitigate anxiety across diverse demographic and cultural settings.

This study revealed that anxiety triggers were culturally and regionally specific, reflecting societal and healthcare influences on patient psychology. In Western countries, anxiety is often attributed to waiting times, fear of pain, and loss of control (Badner et al. 1990), while in countries such as Ethiopia, Pakistan, and Nigeria, concerns about family, mishaps, death, and financial impact are more prevalent (Akinsulore et al. 2015; Nigussie et al. 2014; Jawaid et al. 2007). The findings of this study were mixed, with postoperative pain being the predominant concern as in Western countries, followed by fear of surgical results and concern about family as in Eastern countries. It is also noteworthy that patients in all studies generally prioritized immediate concerns such as pain, complications, and recovery over long-term issues such as financial impact.

## Associations of sociodemographic variables with preoperative anxiety, reasons for feeling anxious, information requirements, and pain levels

1. **Gender**—Previous research has shown that females experience greater preoperative anxiety than males, as demonstrated by a meta-analysis (Semagn Mekonnen et al. 2020), which is attributed to hormonal changes associated with menstrual cycles in women (Iftikhar 2002). However, some studies have found no significant sex differences in preoperative anxiety (Nigussie et al. 2014; Ebirim 2010). In our study, females consistently exhibited slightly greater mean anxiety across trait anxiety levels and all three time-points, with statistically significant difference occurring only at S2. Therefore, gender should not be overly emphasized as an anxiety risk factor. A personalized approach is recommended, as males may experience anxiety but might be less likely to report it due to societal norms that often encourage women more than men to express their emotions.
2. **Age**—Our study showed that younger individuals exhibited statistically significant higher preoperative and trait anxiety scores, consistent with literature suggesting anxiety decreases with age (Jafar and Khan 2009; Kindler 2000; Iftikhar 2002). However, a few have shown that age is not a determinant of preoperative anxiety (Moerman et al. 1996). These findings of our study can be explained by the fact that younger individuals are more concerned about the implications of surgery on their life trajectories, including physical abilities, career development, and personal plans. However, older individuals often exhibit better emotional regulation and greater acceptance of medical interventions with age. Additionally, younger age did not correlate with higher postoperative anxiety or pain levels. People in the > 50-year-old group had higher postoperative anxiety scores than preoperative anxiety scores. This finding explains the other finding from this study, where past medical history (18.00 vs. 15.67, p value—0.037), and higher pain levels were positively correlated (Pearson correlation – 0.4, p value – 0.001) with statistically significantly higher S3 scores. Older people have more chronic ailments and complex surgeries than younger individuals which contributes to their longer duration of surgeries causing higher pain postoperatively, which causes higher postoperative S3 scores than younger individuals, as shown in Table 2. Caumo et al. also reported that a higher ASA grade was associated with a higher number of anxious patients (Caumo et al. 2001).
3. **Marital status**—Unmarried individuals exhibited statistically significantly higher levels of trait anxiety and preoperative anxiety, i.e., S1 and S2. This phenomenon may be attributed to age, as married individuals were generally older than their unmarried counterparts. Additionally, marriage can provide emotional and financial support from one's partner, and the responsibility and focus on family well-being in a committed relationship may redirect attention from personal anxieties, potentially contributing to anxiety reduction. Conversely, unmarried persons may experience societal pressure and validation, which may contribute to elevated anxiety levels.
4. **Education**—An inverse trend was observed in anxiety scores with increasing education levels, although the difference was not statistically significant. Existing literature presents conflicting findings, with some studies indicating higher anxiety levels in lower education groups and others suggesting the opposite (Nigussie et al. 2014; Jafar and Khan 2009). The findings in our study may stem from communication barriers that hinder understanding and amplify fears in lower education groups (Mulugeta et al. 2018; Prathapan n.d.; Wondmieneh 2020). Conversely, graduates possessing enhanced critical thinking skills and access to information are more likely to have substantive interactions with healthcare providers, thereby gaining a more comprehensive understanding of the surgical process and thus experiencing lessanxiety.
5. **Occupation**—Unemployed and skilled workers exhibited the highest preoperative anxiety preoperatively, but this was statistically significant only in S2. This can be attributed to the group stratification, which includes females and young in the unemployed category, and the lower education group in the skilled worker category, which were observed to have feelings of being inadequately informed and higher needs of information in our study. Semi-skilled workers showed the most substantial decrease in anxiety levels from S1 (20.20) to S3 (15.6). Conversely, professionals showed an increase in anxiety levels from S1 (17.81) to S3 (18.05). In addition, the professionals displayed the highest postoperative anxiety among the other groups, which was not statistically significant. This might have originated from concerns about the loss of control and the potential impact on their professional identity during the recovery period.
6. **Past surgical history (past anesthesia exposure)**—Our study demonstrated consistently lower anxiety levels among patients with previous surgical experience, although the difference was not statistically significant, a finding corroborated by several other studies (Nigussie et al. 2014; Jawaid et al. 2007). However, some related research shows a significant relationship, which may reflect varying patient experiences in varied healthcare settings (Mulugeta et al. 2018; Matthias et al. 2012). Patients with positive past surgical experiences may exhibit lesser anxiety, while those with negative experiences may exhibit more anxiety, potentially accounting for the discrepancy in results across various studies.
7. **Type of anesthesia**—Several studies have indicated that general anesthesia is linked to higher levels of anxiety than spinal anesthesia, possibly due to the loss of a sense of control and more complex surgeries associated with general anesthesia (Pokharel et al. 2011; Mitchell and Mitchell 2013). However, our study did not demonstrate a statistically significant correlation in this regard, although a similar trend was observed as in other investigations (Woldegerima Berhe et al. 2022).
8. **Pain**—Multiple studies have reported a relationship between higher preoperative anxiety scores and greater postoperative pain levels (Fonseca-Rodrigues et al. 2021). However, our study demonstrated that postoperative pain levels are associated with only postoperative anxiety, that is, STAI S3. This variation may be attributed to our sample, which included younger participants who underwent less complex surgeries of shorter durations. In our study, shorter surgical procedures correlated with lower postoperative pain levels, resulting in greater satisfaction and consequently, reduced postoperative anxiety from higher preoperative anxiety. Regarding postoperative anxiety, Ghoneim et al. reported that unresolved postoperative anxiety can lead to long-term psychiatric conditions, higher infection rates, and tumor progression, ultimately increasing morbidity and mortality after surgery (Ghoneim et al. 2016). Therefore, managing postoperative anxiety is as crucial as addressing preoperative anxiety, emphasizing the need for screening and early management of such individuals.

## Information requirements

Our research indicated that approximately 25–30% of patients felt inadequately informed about their surgery and anesthesia, and having more information would have helped them feel more relaxed. Individuals who were under-informed about surgery exhibited a significantly higher level of anxiety, whereas no significant correlation was observed for those under-informed about anesthesia. This finding aligns with one study wherein patients who lacked information about surgery demonstrated higher state anxiety levels, while knowledge about anesthesia did not influence state anxiety levels (Kiyohara et al. 2004). The overall trend toward higher anxiety scores in individuals who perceived that more information about surgery and anesthesia would be beneficial for relaxation, although not always statistically significant, indicates that information delivery is a crucial aspect of anxiety management but cannot completely mitigate anxiety levels, suggesting the need for additional interventions. Younger individuals are more likely to report that additional information about surgery would contribute to their relaxation compared to older patients. This finding is consistent with a study conducted in the Nepalese population, in which age < 30 years was identified as an independent factor for the need to seek information about surgery and anesthesia during the preoperative period (Pokharel et al. 2011). Other studies have proposed that anxiety decreases with age due to the fatalistic view of elderly patients, which leads to reduced uncertainty and less information-seeking (Celik et al. 2018; Aykent et al. 2007). Additionally, younger individuals may have less experience with health-related procedures and associated stress, rendering the prospect of surgery more anxiety-provoking due to the fear of the unknown or fear of pain. In conclusion, there are differences in the information requirements across sociodemographic groups, which emphasizes the need for personalized interviewing to understand individual information requirements to prevent any paradoxical increase in anxiety due to inappropriate information delivery.

### Limitations

This study presents several limitations. First, the effects of various surgeries on anxiety levels were not differentiated. Furthermore, the study was constrained by a limited sample size, which restricted the ability to investigate variables such as alcohol use, smoking history, and illicit substance use due to uneven distribution among the study participants.

## Conclusion

Anxiety levels were observed to be highest on the day of surgery, lowered in the preoperative period, and further decreased postoperatively. The identified risk factors included younger age, unmarried status, skilled occupation, and female gender, while older age and a past medical history contributed to postoperative anxiety. Premedication alone was insufficient, indicating the need for additional strategies. Moreover, 25–30% of patients reported feeling inadequately informed about surgery and anesthesia, with higher information requirements about surgery correlating with the higher anxiety levels. A disparity in information-seeking behavior was noted among different sociodemographic groups, with younger patients expressing a more desire for additional surgical information to alleviate anxiety.

## Supplementary Information

Additional file 1. State-Trait Anxiety Inventory for Adults Short Form Scoring Key.

Additional file 2. Perioperative questionnaire.

## Data Availability

All data generated or analyzed during this study were included in this published article [and its supplementary information files].
