# Supplementary material for: Assessment of perioperative anxiety levels at three time-points during hospital stay in patients undergoing elective surgery
Source: Perioper Med (Lond). 2025 Mar 12;14:27. doi: 10.1186/s13741-025-00504-0 (PMC11905583; doi:10.1186/s13741-025-00504-0)
Supplement: Supplementary file 1 — Additional file 1. State-Trait Anxiety Inventory for Adults Short Form Scoring Key. [file 13741_2025_504_MOESM1_ESM.pdf]

For use by Priya Goyal only. Received from Mind Garden, Inc. on July 11, 2023

State-Trait Anxiety Inventory for Adults Short Form Scoring Key
(Short Form Y-1, Short Form Y-2)

Developed by Charles D. Spielberger in collaboration with R.L. Gorsuch, R. Lushene, P.R. Vagg, and G.A. Jacobs

To use this stencil, line up with the appropriate test side, either Short Form Y-1 or Short Form Y-2. Simply total the scoring **weights** shown on the stencil for each response category. For example, for question # 1, if the respondent marked 3, then the **weight** would be **2**. Refer to the State Trait Anxiety Inventory for Adults manual for appropriate normative data.

Short Form Y-1

		NOT AT ALL	SOMEWHAT	MODERATELY SO	VERY MUCH SO
**1.**	**I feel calm**	**4**	**3**	**2**	**1**
2.	I am tense	1	2	3	4
**3.**	**I feel at ease**	**4**	**3**	**2**	**1**
4.	I am presently worrying over possible misfortunes	1	2	3	4
5.	I feel frightened	1	2	3	4
6.	I feel nervous	1	2	3	4
7.	I am jittery	1	2	3	4
**8.**	**I am relaxed**	**4**	**3**	**2**	**1**
9.	I am worried	1	2	3	4
**10.**	**I feel steady**	**4**	**3**	**2**	**1**

STAIAD instrument © 1968, 1977 Charles D. Spielberger. All rights reserved in all media.
Published by Mind Garden, Inc., www.mindgarden.com

For use by Priya Goyal only. Received from Mind Garden, Inc. on July 11, 2023

Short Form Y-2

		ALMOST NEVER	SOMETIMES	OFTEN	ALMOST ALWAYS
11.	I feel nervous and restless	1	2	3	4
**12.**	**I feel satisfied with myself**	**4**	**3**	**2**	**1**
13.	I wish I could be as happy as others seem to be	1	2	3	4
14.	I feel like a failure	1	2	3	4
15.	I worry too much over something that really doesn't matter	1	2	3	4
16.	I lack self-confidence	1	2	3	4
**17.**	**I feel secure**	**4**	**3**	**2**	**1**
18.	I feel inadequate	1	2	3	4
**19.**	**I am a steady person**	**4**	**3**	**2**	**1**
20.	I get in a state of tension or turmoil as I think over my recent concerns and interests	1	2	3	4

STAIAD instrument © 1968, 1977 Charles D. Spielberger. All rights reserved in all media.
Published by Mind Garden, Inc., www.mindgarden.com
