# Supplementary material for: Assessment of perioperative anxiety levels at three time-points during hospital stay in patients undergoing elective surgery
Source: Perioper Med (Lond). 2025 Mar 12;14:27. doi: 10.1186/s13741-025-00504-0 (PMC11905583; doi:10.1186/s13741-025-00504-0)
Supplement: Supplementary file 2 — Additional file 2. Perioperative questionnaire. [file 13741_2025_504_MOESM2_ESM.pdf]

PRE-OPERATIVE QUESTIONNAIRE

1. **MRD. No.** : ………………………………
2. **Name** : ………………………………
3. **Gender** : Male ? Female ? Others ?
4. **Age** : ………………………………
5. **Marital Status** : ………………………………
6. **Residence** : City ? Village ? Town ?
7. **Education Level**

- Post Graduate/ Professional Degree ?
- Graduate ?
- Higher Sec School (passed Class 12) ?
- High School (Passed Class 10) ?
- Middle School (Passed Class 8) ?
- Literate, less than middle school (< Class 8 pass) ?
- Illiterate ?

8. **Occupation**

- Professional (Doctor, Advocate, Engineer, Professor, College principles) ?
- Semi Professional (High School teachers, College Lecturers, Junior medical practitioner) ?
- Arithmatic skill jobs (Clerk, Accountant, Typist, Elementary school teacher) ?
- Skilled Worker (Driver, Mason, Carpentor, Mechanic) ?
- Semi Skilled worker (Factory labourer, car cleaner, small shopkeeper) ?
- Unskilled (Domestic Servant, Peon, Watchman) ?
- Unemployed ?

9. **Past Medical History:** ……………………………
10. **Past Surgical History**: ……………………………
12. **Current Surgery**: ……………………………
13. **Smoking history :** ……………………………
14. **Alcohol history:** ……………………………..
15. **Drug history:** ……………………………

16. Does any of the following is currently bothering you?

1. Fear of Feeling pain during surgery ?
2. Fear of results of surgery ?
3. Fear of feeling pain after surgery ?
4. Waking up during surgery ?
5. Fear of complications ?
6. Concern about family ?
7. Financial burden ?
8. Fear of unknown ?
9. Others ?
10. None ?

POST-OPERATIVE QUESTIONNAIRE

MRD No.:………… **Time since operation**……………

1. Do you think you were given adequate information about anesthesia?

Yes ? No ?

2. Do you think if you were more information about anesthesia, it would have made you more released?

Yes ? No ?

3. Do you think you were given adequate information about surgery?

Yes ? No ?

4. Do you think if you were given more information about surgery, it would have made you more relaxed?

Yes ? No ?
